# Reverse Shoulder Prosthesis for Proximal Humeral Fractures: Primary Treatment vs. Salvage Procedure

**DOI:** 10.3390/jcm13113063

**Published:** 2024-05-23

**Authors:** Antonio Caldaria, Luca Saccone, Nicolò Biagi, Edoardo Giovannetti de Sanctis, Angelo Baldari, Alessio Palumbo, Francesco Franceschi

**Affiliations:** 1 Department of Orthopaedic and Trauma Surgery, San Pietro Fatebenefratelli Hospital, 00189 Rome, Italy; acaldaria@gmail.com (A.C.);; 2 Faculty of Medicine and Surgery, UniCamillus-Saint Camillus International University of Health and Medical Sciences, 00131 Rome, Italy; 3 Department of Orthopaedics and Traumatology, Fondazione Policlinico Universitario Campus Bio-Medico of Rome, 00128 Rome, Italy; 4 Informatics Research Centre, Business Informatics Systems and Accounting, Henley Business School, University of Reading, Reading RG9 3AU, UK; nico.biagi@gmail.com; 5 Institut Universitaire Locomoteur et du Sport (IULS), Hôpital Pasteur 2, CHU de Nice, 30, Avenue Voie Romaine, 06000 Nice, France

**Keywords:** proximal humeral fractures, reverse total shoulder arthroplasty, fractures sequelae, salvage shoulder arthroplasty

## Abstract

**Background:** The optimal treatment for complex proximal humerus fractures (PHFs) lacks consensus, with reverse total shoulder arthroplasty (RTSA) often being a final resort rather than a primary approach. This study aimed to compare outcomes and satisfaction rates of primary RTSA for PHFs versus salvage RTSA for previously unsuccessful treatments. We hypothesized that primary RTSA would yield superior clinical outcomes, functional scores, and patient satisfaction. **Methods:** A retrospective analysis of RSA procedures between 2011 and 2021 was conducted, focusing on primary RTSA for PHFs or salvage RTSA for failed osteosynthesis. Patients meeting inclusion criteria underwent clinical and radiological follow-up for at least two years. Demographic characteristics, outcomes scores, and range of motion (ROM) were assessed. **Results:** Of 63 patients, 42 underwent primary RTSA and 21 underwent salvage RTSA. The median follow-up was 50 months. Statistically significant differences favored primary RTSA in forward flexion, abduction, internal rotation, and Constant shoulder score. Patient satisfaction levels did not significantly differ between groups. Complications occurred in 7.15% of primary RTSA cases and 14.28% of salvage RTSA cases. **Conclusions:** Primary RTSA may yield slightly better outcomes and lower complication rates compared to salvage RTSA. Further prospective studies are necessary to validate these findings.

## 1. Introduction

**Figure 1:**
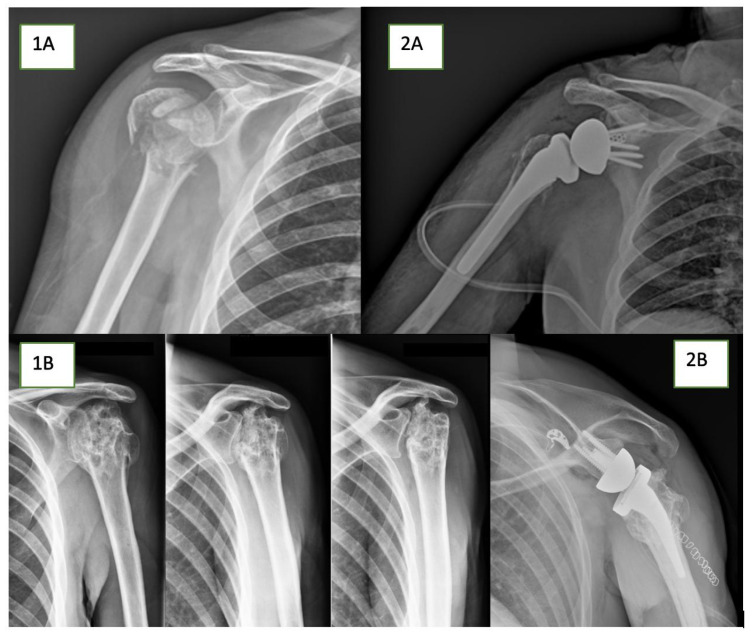
A four-part PHF (**1A**) treated with primary RTSA (**2A**). Fracture sequelae with cephalic collapse (**1B**) treated with salvage RTSA (**2B**).

A recent epidemiological study [1] has highlighted that the incidence of proximal humerus fractures (PHF) is 110 per 100,000/person-year. This incidence is 1.7 times higher compared to the one considering only inpatient cases (66.5 per 100,000/person-year). From these data, it can be inferred that only 40% of all patients with PHF are not compelled to undergo hospitalization for necessary care. For patients older than 65 years old (the population most affected by PHF), the nonoperative management rate is reported at 85% [2]. Such a high percentage of nonsurgical indications could be easily interpreted considering that different studies showed no significant differences between surgical treatment compared with nonsurgical treatment in patient-reported clinical outcomes in the case of displaced PHF involving the surgical neck [3,4]. Despite this data, there is an increasing trend in surgery for patients with PHF [3], mainly due to a lack of a universally agreed-upon consensus regarding the optimal treatment for complex PHFs [5]. Focusing on surgical indications, interesting considerations can be made by evaluating the data in the contemporary scientific literature. Patel et al. [2], utilizing the Cochran–Armitage trend test collecting data from 2010 to 2019 of surgical treatments for PHF, observed that Open Reduction and Internal Fixation (ORIF) procedures have decreased by 25.7%, intramedullary nailing (IMN) procedures have decreased by 81.9%, hemiarthroplasty (HA) procedures have decreased by 81.4%, total shoulder arthroplasty (TSA) have decreased by 80.5%, and reverse total shoulder arthroplasty (RTSA) procedures have increased by 1841.4%. Several reasons explain this trend. ORIF and IMN procedures are associated with frequent complications, including screw cutout, a dysfunctional shoulder due to nonhealing of the tuberosities and glenoid erosion, avascular necrosis of the humeral head, malunion, and nonunion [6,7,8]. By evaluating HA and TSA surgeries instead, the need to perform anatomic reconstruction of the tuberosities is well established: different studies have correlated the clinical outcomes and survival rate of these surgical treatments with the need to have good tuberosities and so a competent rotator cuff [9,10]. Among this evolving spectrum of treatment options, RTSA has emerged as a viable solution, both as a primary intervention for PHF and in cases of failed other treatments [5,11] (Figure 1). The biomechanical properties of RTSA [12,13], characterized by its semi-constrained design and decreased dependence on the union of tuberosities and rotator cuff function, theoretically provide an advantage over traditional nonconstrained arthroplasty in instances of unsuccessful internal fixation [14]. However, the potential impact of previous surgery on the feasibility of future salvage RTSA remains uncertain. The purpose of this study was to compare the outcomes and satisfaction rate of primary RTSA for PHF versus salvage RTSA for failed previous osteosynthesis. Our hypothesis is that patients undergoing primary RTSA will exhibit superior clinical outcomes, functional scores, and levels of patient satisfaction compared to those undergoing salvage RTSA.

## 2. Material and Methods

### 2.1. Study Design

In our tertiary referral hospital, we conducted a retrospective analysis of a consecutive series of RTSA procedures performed between 2011 and 2021. From these procedures, we selected those performed as either primary treatment for PHF or salvage treatment for previously unsuccessful osteosynthesis. During this period, 42 patients underwent a primary RTSA acutely, and 21 underwent RSA after a prior failed osteosynthesis of a proximal humeral fracture. A total of 14 patients had osteosynthesis using plate and screws, 6 patients were primarily treated with IMN, and 1 patient was treated with transcutaneous K-wire fixation. The rationale for performing RTSA in all patients with prior osteosynthesis was the presence of pain and restricted functionality. The painful dysfunction was associated with post-traumatic degenerative joint disease (n = 10, 47.6%), screw cutout (n = 6, 28.6%), humeral head osteonecrosis with collapse (n = 3, 14.3%), and malunion (n = 2, 9.5%). No cases of failed osteosynthesis were attributable to infection. There were no pre-operative indications of peri-implant joint infection in any of the patients, and all routine tests performed during surgery, such as peri-implant tissue cultures and sonication of the implant materials, produced negative results. CT scans and standard anteroposterior, axillary lateral, and scapular radiographs were used for preoperative planning and to classify fracture sequelae according to Boileau classification [15].

### 2.2. Inclusion and Exclusion Criteria

The inclusion criteria comprised patients initially treated with ORIF or fixation using an IMN or transcutaneous K-wire fixation and subsequently managed with a single-stage revision to RTSA, patients treated with primary RTSA for proximal humerus fractures, and patients who underwent a minimum of two years of clinical and radiological follow-up. The exclusion criteria comprised patients who did not follow the rehabilitation protocol as recommended, patients who experienced iatrogenic fractures during RTSA surgery, and polytrauma patients. 

### 2.3. Data Extraction

The clinical outcomes were evaluated using the Constant–Murley Shoulder Outcome Score (CS) and a zero to ten visual analog scale (VAS) for pain. Patient satisfaction with the procedure was also self-rated as excellent, good, satisfactory, poor, or unsatisfactory. Range of motion (ROM), encompassing forward flexion, abduction, internal rotation, and external rotation at 0° and 90° of shoulder abduction, was documented at the final follow-up. Internal rotation was measured as the highest vertebral level reached by the thumb. The postoperative rehabilitation protocol was similar for both patient groups. Immediately after surgery, the arm was secured in an abduction brace for a duration of 4 weeks. Pendulum exercises and passive mobilization of the shoulder joint were initiated on the first day. Following the initial 4-week period, the brace was removed, and a gradual introduction of active-assisted shoulder mobilization was commenced.

### 2.4. Statistical Analysis

Statistical analysis was conducted using R (R Core Team, 2020, Vienna, Austria), RStudio (Rstudio Team, 2020, Boston, MA, USA), and the Rstatix package (Rstudio Team, 2023, Boston, MA, USA). In all tests, statistical significance was considered for *p* < 0.05. The parametric and nonparametric distribution was determined using the Shapiro–Wilk and D’Agostino–Pearson tests. For comparisons between groups, the unpaired Student’s t test was used for parametric data, and the Wilcoxon Rank-Sum test was used for nonparametric continuous variables. The X2 test, Fisher’s exact test, was used to analyze categorical variables.

### 2.5. Surgical Technique

The senior author, FF, conducted all procedures with the patient in a beach-chair position under general anesthesia combined with an interscalene block. The procedures were conducted in a room equipped with laminar flow. A standard deltopectoral approach was executed with the arm secured in a limb holder (TRIMANO, Arthrex Inc., Naples, FL, USA). The procedures were performed utilizing four distinct implant constructs: Aequalis Ascend Flex Reverse System 145° (Stryker Corp., Kalamazoo, MI, USA), Equinoxe Reverse 145° (Exactech, Inc.; Gainesville, FL, USA), Univers Reverse 135° (Arthrex Inc., Naples, FL, USA), and Comprehensive Shoulder Arthroplasty System (Zimmer Biomet, Warsaw, IN, USA). Each system was implemented following standardized procedures outlined in the respective manufacturers’ technical manuals. Following preoperative planning based on CT studies, the glenoid components were positioned with an inferior inclination in order to correct the reverse shoulder angle [16] and to achieve an inferior overhang of approximately 3 mm. The retroversion of the glenoid component was maintained within a range of −5 to −10 degrees. The humeral component was consistently placed with 20 degrees of retroversion. The biceps tendon, if present, underwent tenodesis. In severe malunions and primary RTSA, the tuberosities were osteotomized and reattached to the implant and humeral shaft, aiming for placement as close as possible to their original anatomic site. The management of the tuberosities followed the techniques described by Boileau et al. [17]. The height of the prosthetic stem was established by utilizing the contralateral humerus as a template [18], with the objective of achieving an overall humeral lengthening ranging from 2 to 2.5 cm relative to the unaffected side. In cases where the contralateral humerus could not be used as a reference due to factors like previous trauma altering the anatomy or prior RTSA, the height of the humeral stem was determined using the following criteria: (1) the height of the fractured tuberosities, (2) the insertion points of the deltoid and pectoralis major tendon, (3) the measurement of the cartilage-free zone of the humeral head at the calcar, which defines the medial-inferior border of the prosthetic head, and (4) the measurement from the tip of the fractured greater tuberosity to the articular-side insertion of the rotator cuff, indicating the height of the lateral aspect of the humeral head. The trial humeral stem was momentarily placed at the desired height by blocking it with a sterile gauze inserted into the canal. At this stage, the stability of the implant was evaluated, and if judged satisfactory, the final implant was positioned, and the tuberosities were synthesized. The decision to use cementation for the stem was made intraoperatively, based on considerations of bone density and the effectiveness of achieving press-fit engagement with the largest feasible stem. Using a press-fit humeral stem in PHF with 3 or 4 displaced fragments is exceedingly uncommon in our routine surgical treatment. In fact, all the implants employed in the study featured a cemented humeral stem. We used cement restrictors into the canal and a medium viscosity, radiopaque bone cement containing gentamicin.

### 2.6. Ethical Approval

This study has been reviewed and granted ethical approval by the Ethics Committee of UniCamillus-Saint Camillus International University of Health Sciences, Rome, Italy. The research methodology, including data collection, participant recruitment, and any associated procedures, has been designed and conducted with careful consideration of ethical principles. Measures have been implemented to ensure the protection of participants’ rights, confidentiality, and welfare throughout the duration of the study. All participants involved in the research project have been provided with clear and comprehensive information regarding the study objectives and procedures.

## 3. Results

**Figure 2:**
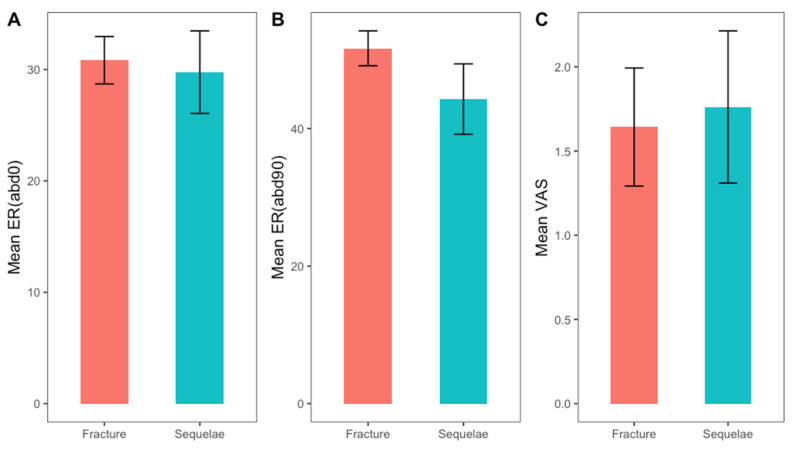
Bar plots for external rotation at 0° (**A**) and 90° (**B**) and visual analog scale (VAS) (**C**). All plots include mean value and relative effect size for the two groups.

**Figure 3:**
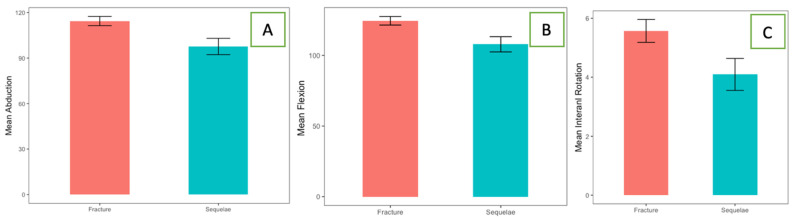
Bar plots for abduction° (**A**), anterior forward flexion (**B**), and internal rotation (**C**). All plots include mean value and relative effect size for the two groups.

**Figure 4:**
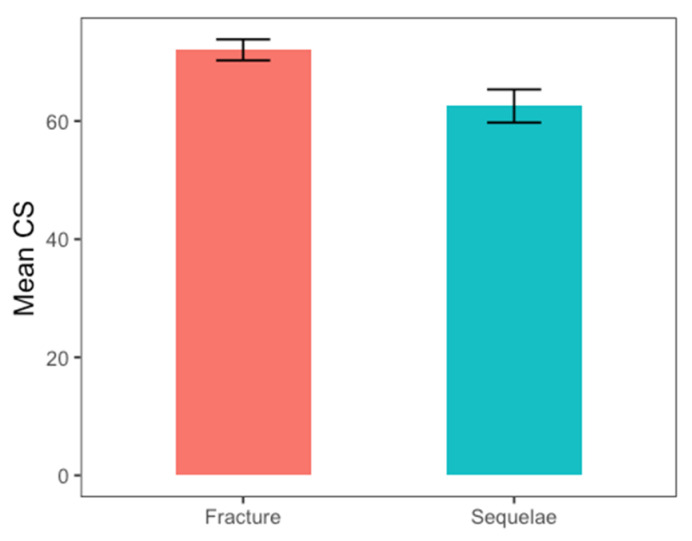
Bar plots for Constant score (CS) with mean value and relative effect size for the two groups.

There were 46 women and 17 men. A total of 42 patients underwent a primary RTSA acutely, and 21 underwent RSA after a prior failed ORIF of a proximal humeral fracture. The mean age was 73 years old (range 42−87 years old). No significant differences were found in the demographic characteristics between the two groups (Table 1). The median follow-up time was 50 ± 22.8 months in both groups. The mean time from fracture to primary arthroplasty was 5 days, while the mean time from osteosynthesis to secondary arthroplasty was 15 months. The outcomes scores and ROM are summarized in Table 2. When the salvage RTSA cohort was compared with the primary RTSA cohort, no significant differences were observed in external rotation at 0° (W = 467.5; *p*-value = 0.7) and 90° of abduction (t = 1.45; *p*-value = 0.15) and VAS (W = 406; *p*-value = 0.59; Figure 2). Statistically significant differences have been observed in anterior forward flexion (W = 598; *p*-value = 0.02; the effect size was small r = 0.29), abduction (W = 624.5; *p*-value = 0.007, the effect size was moderate r = 0.4), internal rotation (W=574.5; *p*-value = 0.046; the effect size was small r = 0.25; Figure 3), and CS (Figure 4; W = 620; *p*-value = 0.009; the effect size was moderate r = 0.33). Despite the better outcomes observed in the primary RTSA cohort, no statistically significant differences were observed in the level of patient satisfaction (*p*-value = 0.79). Post hoc power analyses were calculated in order to obtain the achieved power. For the unpaired Student t test, an observed power of 0.4528636 was achieved, while for the nonparametric Wilcoxon Rank-Sum test, an observed power of 0.4357446 was achieved. We observed three complications (7.1%) in the primary RTSA cohort and three complications in the salvage RTSA cohort (14.3%). The types of complications are shown in Table 3. Two complications in the primary RTSA cohort required revision surgery.

## 4. Discussion

The findings in our study suggest that patients with PHF can achieve notably improved ROM and enhanced patient-reported clinical scores when opting for primary RTSA compared to salvage RTSA. Our results suggest that salvage RTSA consistently yields durable outcomes in terms of clinical function and pain relief for individuals with challenging cases of painful and severely impaired shoulders, where alternative treatment approaches have demonstrated limited success. Our results show favorable outcomes in both groups regarding range of motion, postoperative pain measured using the VAS scale, and clinical scores. We observed a statistically significant difference favoring primary RTSA in terms of abduction, anterior forward flexion, internal rotation, and clinical scores. No statistically significant differences were noted in external rotation, VAS score, or patient satisfaction levels between the two groups. Several studies in the literature have investigated the outcomes of different treatment options for PHFs. Shannon et al. [19] demonstrated that RTSA performed after unsuccessful ORIF demonstrated a slightly elevated incidence of complications (8%) compared to primary RTSA (5%), but the rates of revision and reoperation, along with clinical outcomes and shoulder function, remained similar during the initial follow-up period. There were no significant differences observed in postoperative clinical scores between the two groups. Similarly, there were no notable distinctions in shoulder elevation (130° vs. 133°, *p*-value = 0.785) or external rotation (42° vs. 36°, *p*-value = 0.51). Dezfuli et al. [20] demonstrated that the group undergoing primary RTSA exhibited notably superior clinical scores compared to the salvage RTSA group and a significantly better active ROM in external rotation (*p*-value < 0.031). However, no discernible disparity was observed between the groups in terms of forward elevation, abduction, and internal rotation. In the study conducted by Sebastia-Forcada et al. [21], the mean clinical scores were significantly lower in the group with salvage procedures compared to those in the primary RTSA group. However, there were no significant differences observed in postoperative mean VAS-pain scores (*p*-value = 0.927), and VAS-satisfaction scores showed no significant variation between the two groups (*p*-value = 0.109). The primary RTSA group exhibited a notably better active range of motion (ROM) in anterior forward flexion and abduction compared to the salvage RTSA group. Seidl et al. [22] demonstrated that both RTSA procedures can lead to favorable clinical outcomes and that primary RTSA typically results in enhanced external rotation motion and a reduced incidence of complications. There were no significant differences in anterior forward flexion and evaluated clinical scores between the two groups. Katthaghen et al. [23] showed that the primary RTSA group had superior clinical shoulder function compared to salvage RTSA, with significantly better abduction, adduction, and forward flexion. However, there were no significant differences observed in external and internal rotation between the two groups. A higher incidence of complications was noted following salvage RTSA (complication rate 8/23 = 34.8%) compared to primary RTSA (complication rate 2/28 = 7.1%), with a significant difference observed (*p*-value = 0.013). Additionally, revision surgery was required significantly more often following salvage RTSA (5/23 = 21.7% in 5 patients) compared to primary RTSA (*p*-value = 0.045). These results collected from the contemporary scientific literature exhibit considerable heterogeneity and frequently diverge from each other. For instance, Shannon [19], Katthaghen [23], Kulhmann [24], and Seidl [22] found no statistically significant differences in the analyzed scores between the two groups, whereas Dezfuli [20] and Sebastia-Forcada [21] noted a statistically significant difference favoring primary RTSA. Similar considerations apply to the ROM results analyzed between the two groups. Our results appear good in both groups in terms of range of motion and residual postoperative pain assessed with the VAS scale and CS. Nonetheless, we identified a statistically significant difference in favor of primary RTSAs concerning abduction, anterior forward flexion, internal rotation, and CS. No statistically significant differences were observed in terms of external rotation, VAS score, and level of patient satisfaction. The variations observed among the different studies discussed could be attributed to differences in the types of implants utilized and their respective placements. Did they lateralize glenoid components? Did they correct the reverse shoulder angle? Did they correct the glenoid version? All these variables contribute to the challenge of comparing the results. However, we tried to give some insights into the results contrary to our initial hypothesis. We propose that the absence of a statistically significant disparity in both the VAS scale and satisfaction with the surgical procedure may be caused from a factor intricately tied to the patients’ psychophysical sphere. Patients who undergo salvage RTSA often suffer a painful and functionally compromised limb due to the failure of previous attempts at osteosynthesis. Consequently, despite the inferior functional outcomes observed during the latest follow-up, these patients exhibited VAS scale values and satisfaction levels with the surgical procedure akin to those undergoing primary RTSA. Conversely, the latter group, despite boasting superior functional and clinical outcomes at the latest follow-up, reported lower satisfaction levels. This is attributed to their previous state of well-being prior to the trauma, having never experienced a hypofunctional or notably “altered” limb compared to their healthy contralateral side. Regarding the lack of a statistically significant difference in the extra-rotation movement between the two groups, we attribute this finding to the challenge of adequately addressing the greater tuberosity [17], the real trial inherent in this type of procedure. In our clinical experience, a primary indication to perform a RTSA for PHF is the identification, through pre-operative CT evaluation, of tuberosities significant compromise. This event usually leads to an inability to achieve satisfactory osteosynthesis. In patients considered for salvage RTSA, the results of prior surgeries often compromise the integrity of the tuberosities. Consequently, in both scenarios, during joint replacement, we must manage a similar situation of compromised tuberosities.

### Strengths and Limitations

This study has several strengths, notably that all the surgical procedures were performed by a single skilled shoulder surgeon (FF). Furthermore, the homogeneity of demographic characteristics across both groups adds another layer of strength. Moreover, our study extends beyond the conventional evaluation of patient outcomes solely in terms of range of motion, incorporating an assessment of patient satisfaction. This comprehensive approach reveals that reverse shoulder prostheses, employed following failed osteosynthesis, not only serve as a salvage option but also ensure patient satisfaction comparable to those used initially for PHF. Several limitations are inherent in this study. It is a retrospective study with a relatively small patient cohort, which may introduce biases typical of such a design. Additionally, the use of prosthetic implants of different brands contributes to the heterogeneity of the sample analyzed, although we emphasize that the primary surgeon is familiar with all of these. Furthermore, our study was constrained by limited preoperative data, leading us to focus solely on final follow-up data for analysis. A final limitation of this study is the heterogeneity within the secondary RTSA group, which encompassed patients previously treated by one of three modalities: ORIF, IMN, and transcutaneous K-wire fixation.

## 5. Conclusions

PHFs are one of the most common types of fractures encountered. ORIF is still considered the routine surgical treatment, with salvage arthroplasty reserved in cases of treatment failure. More recently, RTSA has gained popularity as a primary intervention for addressing PHF with three or four fragments [25]. However, there is currently limited data available regarding the outcomes of RTSA used as a salvage procedure following failed osteosynthesis [5]. Our study shows that patients with PHF can attain notably improved ROM and enhanced patient-reported clinical scores when opting for primary RTSA compared to those undergoing salvage RTSA. In case of failed osteosynthesis, salvage RTSA has the potential to provide good and predictable clinical and functional outcomes. However, the clinical implications of these findings remain somewhat uncertain and warrant additional prospective studies involving larger cohorts to validate our outcomes.

## Figures and Tables

**Table 1:** Patient demographics (primary and salvage RTSA, January 2011–January 2021). N°, number; SD, standard deviation.

	Primary RTSA	Salvage RTSA	*p*-Value
Patients (n°)	42	21	
Gender			0.12
Male (n°)	7	8	
Female (n°)	35	13	
Age in years (range)	75.6 (50−87)	72.5 (42−85)	0.09
Surgical site right (n°)	29	11	
Surgical site left (n°)	13	10	
Follow-up in months (Mean ± SD)	51 ± 23.5	49.5 ± 21.8	

**Table 2:** Clinical results. SD, standard deviation. Abd, abduction. VAS, visual analog scale.

	Primary RTSA	Salvage RTSA	*p*-Value
Abduction (Mean ± SD)	114.4° ± 19.6	97.6° ± 24.6	0.007
Anterior forward flexion (Mean ± SD)	124° ± 19.6	107.8° ± 29.4	0.02
Intra rotation (Mean ± SD)	5.6° ± 2.5	4.1° ± 2.5	0.046
External rotation (0° abd) (Mean ± SD)	30.8° ± 13.8	29.8° ± 16.6	0.7
External rotation (90° abd) (Mean ± SD)	51.7° ± 16.4	44.3° ± 23.5	0.15
VAS (Mean ± SD)	1.6° ± 2.3	1.8° ± 2.1	0.59
Constant score (Mean ± SD)	72.1° ± 11.5	62.5° ± 12.9	0.009

**Table 3:** Types of complications. N°, number of patients.

	Primary RTSA	Salvage RTSA
Infection (n°)	1	-
Aseptic glenoid loosening (n°)	1	-
Axillary nerve palsy (n°)	1	1
Heterotopic ossification (n°)	-	1
Thrombosis (n°)	-	1

## Data Availability

The dataset is available on request from the authors.

## References

[1] Koeppe J., Stolberg-Stolberg J., Fischhuber K., Iking J., Marschall U., Raschke M.J., Katthagen J.C. (2023). The incidence of proximal humerus fracture—An Analysis of Insurance Data. Dtsch. Aerzteblatt Online.

[2] Patel A.H., Wilder J.H., Ofa S.A., Lee O.C., Savoie F.H., O’brien M.J., Sherman W.F. (2022). Trending a decade of proximal humerus fracture management in older adults. JSES Int..

[3] Rangan A., Handoll H., Brealey S., Jefferson L., Keding A., Martin B.C., Goodchild L., Chuang L.-H., Hewitt C., Torgerson D. (2015). Surgical vs Nonsurgical Treatment of Adults with Displaced Fractures of the Proximal Humerus: The PROFHER randomized clinical trial. JAMA.

[4] Hohmann E., Keough N., Glatt V., Tetsworth K. (2023). Surgical treatment is not superior to nonoperative treatment for displaced proximal humerus fractures: A systematic review and meta-analysis. J. Shoulder Elb. Surg..

[5] Nelson P.A., Kwan C.C., Tjong V.K., Terry M.A., Sheth U. (2020). Primary Versus Salvage Reverse Total Shoulder Arthroplasty for Displaced Proximal Humerus Fractures in the Elderly: A Systematic Review and Meta-analysis. J. Shoulder Elb. Arthroplast..

[6] Shi X., Liu H., Xing R., Mei W., Zhang L., Ding L., Huang Z., Wang P. (2019). Effect of intramedullary nail and locking plate in the treatment of proximal humerus fracture: An update systematic review and meta-analysis. J. Orthop. Surg. Res..

[7] Lorenz G., Schönthaler W., Huf W., Komjati M., Fialka C., Boesmueller S. (2021). Complication rate after operative treatment of three- and four-part fractures of the proximal humerus: Locking plate osteosynthesis versus proximal humeral nail. Eur. J. Trauma Emerg. Surg..

[8] Sun J.-C., Li Y.-L., Ning G.-Z., Wu Q., Feng S.-Q. (2013). Treatment of three- and four-part proximal humeral fractures with locking proximal humerus plate. Eur. J. Orthop. Surg. Traumatol..

[9] Young A.A., Walch G., Pape G., Gohlke F., Favard L. (2012). Secondary Rotator Cuff Dysfunction Following Total Shoulder Arthroplasty for Primary Glenohumeral Osteoarthritis: Results of a Multicenter Study with More Than Five Years of Follow-up. J. Bone Jt. Surg..

[10] Boileau P., Trojani C., Chuinard C., Lehuec J.-C., Walch G. (2006). Proximal Humerus Fracture Sequelae: Impact of a new radiographic classification on arthroplasty. Clin. Orthop. Relat. Res..

[11] Kimmeyer M., Schmalzl J., Schmidt E., Graf A., Rentschler V., Gerhardt C., Lehmann L.-J. (2024). Surgical treatment of fracture sequelae of the proximal humerus according to a pathology-based modification of the Boileau classification results in improved clinical outcome after shoulder arthroplasty. Eur. J. Orthop. Surg. Traumatol..

[12] Rugg C.M., Coughlan M.J., Lansdown D.A. (2019). Reverse Total Shoulder Arthroplasty: Biomechanics and Indications. Curr. Rev. Musculoskelet. Med..

[13] Hansen M.L., Routman H. (2019). The biomechanics of current reverse shoulder replacement options. Ann. Jt..

[14] Hussey M.M., Hussey S.E., Mighell M.A. (2015). Reverse shoulder arthroplasty as a salvage procedure after failed internal fixation of fractures of the proximal humerus: Outcomes and complications. Bone Jt. J..

[15] Boileau P., Trojani C., Walch G., Krishnan S.G., Romeo A., Sinnerton R. (2001). Shoulder arthroplasty for the treatment of the sequelae of fractures of the proximal humerus. J. Shoulder Elb. Surg..

[16] Boileau P., Gauci M.-O., Wagner E.R., Clowez G., Chaoui J., Chelli M., Walch G. (2019). The reverse shoulder arthroplasty angle: A new measurement of glenoid inclination for reverse shoulder arthroplasty. J. Shoulder Elb. Surg..

[17] Boileau P., Alta T.D., Decroocq L., Sirveaux F., Clavert P., Favard L., Chelli M. (2019). Reverse shoulder arthroplasty for acute fractures in the elderly: Is it worth reattaching the tuberosities?. J. Shoulder Elb. Surg..

[18] Lädermann A., Williams M.D., Melis B., Hoffmeyer P., Walch G. (2009). Objective evaluation of lengthening in reverse shoulder arthroplasty. J. Shoulder Elb. Surg..

[19] Shannon S.F., Wagner E.R., Houdek M.T., Cross W.W., Sánchez-Sotelo J. (2016). Reverse shoulder arthroplasty for proximal humeral fractures: Outcomes comparing primary reverse arthroplasty for fracture versus reverse arthroplasty after failed osteosynthesis. J. Shoulder Elb. Surg..

[20] Dezfuli B., King J.J., Farmer K.W., Struk A.M., Wright T.W. (2016). Outcomes of reverse total shoulder arthroplasty as primary versus revision procedure for proximal humerus fractures. J. Shoulder Elb. Surg..

[21] Sebastia-Forcada E., Lizaur-Utrilla A., Cebrian-Gomez R., Miralles-Muñoz F.A., Lopez-Prats F.A. (2017). Outcomes of Reverse Total Shoulder Arthroplasty for Proximal Humeral Fractures: Primary Arthroplasty Versus Secondary Arthroplasty After Failed Proximal Humeral Locking Plate Fixation. J. Orthop. Trauma.

[22] Seidl A., Sholder D., Warrender W., Livesey M., Williams G., Abboud J., Namdari S. (2017). Early Versus Late Reverse Shoulder Arthroplasty for Proximal Humerus Fractures: Does It Matter?. Arch. Bone Jt. Surg..

[23] Katthagen J.C., Hesse E., Lill H., Schliemann B., Ellwein A., Raschke M.J., Imrecke J. (2020). Outcomes and revision rates of primary vs. secondary reverse total shoulder arthroplasty for proximal humeral fractures. Obere. Extremität..

[24] Kuhlmann N.A., Taylor K.A., Roche C.P., Franovic S., Chen C., Carofino B.C., Flurin P.-H., Wright T.W., Schoch B.S., Zuckerman J.D. (2020). Acute versus delayed reverse total shoulder arthroplasty for proximal humerus fractures in the elderly: Mid-term outcomes. Semin. Arthroplast. JSES.

[25] Holton J., Yousri T., Arealis G., Levy O. (2017). The role of reverse shoulder arthroplasty in management of proximal humerus fractures with fracture sequelae: A systematic review of the literature. Orthop. Rev..

